# Mitochondrial folate pathway regulates myofibroblast differentiation and silica-induced pulmonary fibrosis

**DOI:** 10.1186/s12967-023-04241-0

**Published:** 2023-06-06

**Authors:** Yaqian Qu, Ruonan Zhai, Dandan Wang, Zheng Wang, Guangjie Hou, Chenchen Wu, Meian Tang, Xiongbin Xiao, Jie Jiao, Yue Ba, Fang Zhou, Jian Qiu, Wu Yao

**Affiliations:** 1 Department of Occupational and Environmental Health, College of Public Health, Zhengzhou University, Zhengzhou, Henan China; 2 Hunan Key Laboratory of Molecular Precision Medicine, Department of Neurology, Xiangya Hospital, Central South University, Changsha, Hunan China; 3 Public Health and Preventive Medicine Teaching and Research Center, Henan University of Chinese Medicine, Zhengzhou, Henan China; 4 Hunan Prevention and Treatment Institute for Occupational Diseases, Changsha, Hunan China; 5 Henan Institute for Occupational Health, Zhengzhou, Henan China; 6 Hunan Key Laboratory of Medical Genetics, School of Life Sciences, Central South University, Changsha, Hunan China; 7 National Clinical Research Center for Geriatric Disorders, Xiangya Hospital, Central South University, Changsha, Hunan China

**Keywords:** Silicosis, Oxidative stress, Folate, Mitochondria, MTHFD2, SLC25A32

## Abstract

**Background:**

Silica-induced pulmonary fibrosis (silicosis) is a diffuse interstitial fibrotic disease characterized by the massive deposition of extracellular matrix in lung tissue. Fibroblast to myofibroblast differentiation is crucial for the disease progression. Inhibiting myofibroblast differentiation may be an effective way for pulmonary fibrosis treatment.

**Methods:**

The experiments were conducted in TGF-β treated human lung fibroblasts to induce myofibroblast differentiation in vitro and silica treated mice to induce pulmonary fibrosis in vivo.

**Results:**

By quantitative mass spectrometry, we revealed that proteins involved in mitochondrial folate metabolism were specifically upregulated during myofibroblast differentiation following TGF-β stimulation. The expression level of proteins in mitochondrial folate pathway, MTHFD2 and SLC25A32, negatively regulated myofibroblast differentiation. Moreover, plasma folate concentration was significantly reduced in patients and mice with silicosis. Folate supplementation elevated the expression of MTHFD2 and SLC25A32, alleviated oxidative stress and effectively suppressed myofibroblast differentiation and silica-induced pulmonary fibrosis in mice.

**Conclusion:**

Our study suggests that mitochondrial folate pathway regulates myofibroblast differentiation and could serve as a potential target for ameliorating silica-induced pulmonary fibrosis.

**Supplementary Information:**

The online version contains supplementary material available at 10.1186/s12967-023-04241-0.

## Background

Silicosis is a pulmonary interstitial disease distinguished by chronic fibrosis development and accompanied by progressive pulmonary dysfunction without curative therapy [1–3]. A typical pathological feature of this progressive pulmonary fibrosis is the activation and persistence of myofibroblasts, which are mainly derived from lung fibroblasts under TGF-β stimulation [4–9]. Myofibroblasts are characterized by high expression of alpha smooth muscle actin (α-SMA), strong contraction and migration capabilities, and secretion of large amounts of extracellular matrix (ECM) with typical contents like fibronectin 1 (FN1) and collagen type I alpha 1 (COL1A1) [10–12]. Myofibroblasts play pivotal roles in fibrosis and have been regarded as potential target for developing effective therapeutic strategies to treat lung fibrosis [13, 14]. To meet the energy demands during myofibroblast differentiation, mitochondrial biogenesis is stimulated and metabolic processes are reprogrammed to enhance cellular ATP production to meet the energetic demands [15–17]. While producing vast majority of cellular ATP, mitochondria also function as a major site to generate reactive oxygen species (ROS) [18, 19]. Although low level of ROS plays important roles as ‘physiological necessity’ to regulate a wide range of critical cellular events [20, 21], oxidative stress caused by excessive amounts of ROS are detrimental and can promote myofibroblast differentiation and pulmonary fibrosis [22–25]. To antagonize oxidative stress, antioxidant enzymatic systems, including superoxide dismutases, catalase, glutathione peroxidases and peroxiredoxins, have been developed during evolution to fine tune the intracellular redox balance [26, 27]. Moreover, small molecules such as reduced glutathione and NADPH continuously fuel reducing power to ensure the recycling of antioxidant enzymes in redox homeostasis.

**Fig. 1:**
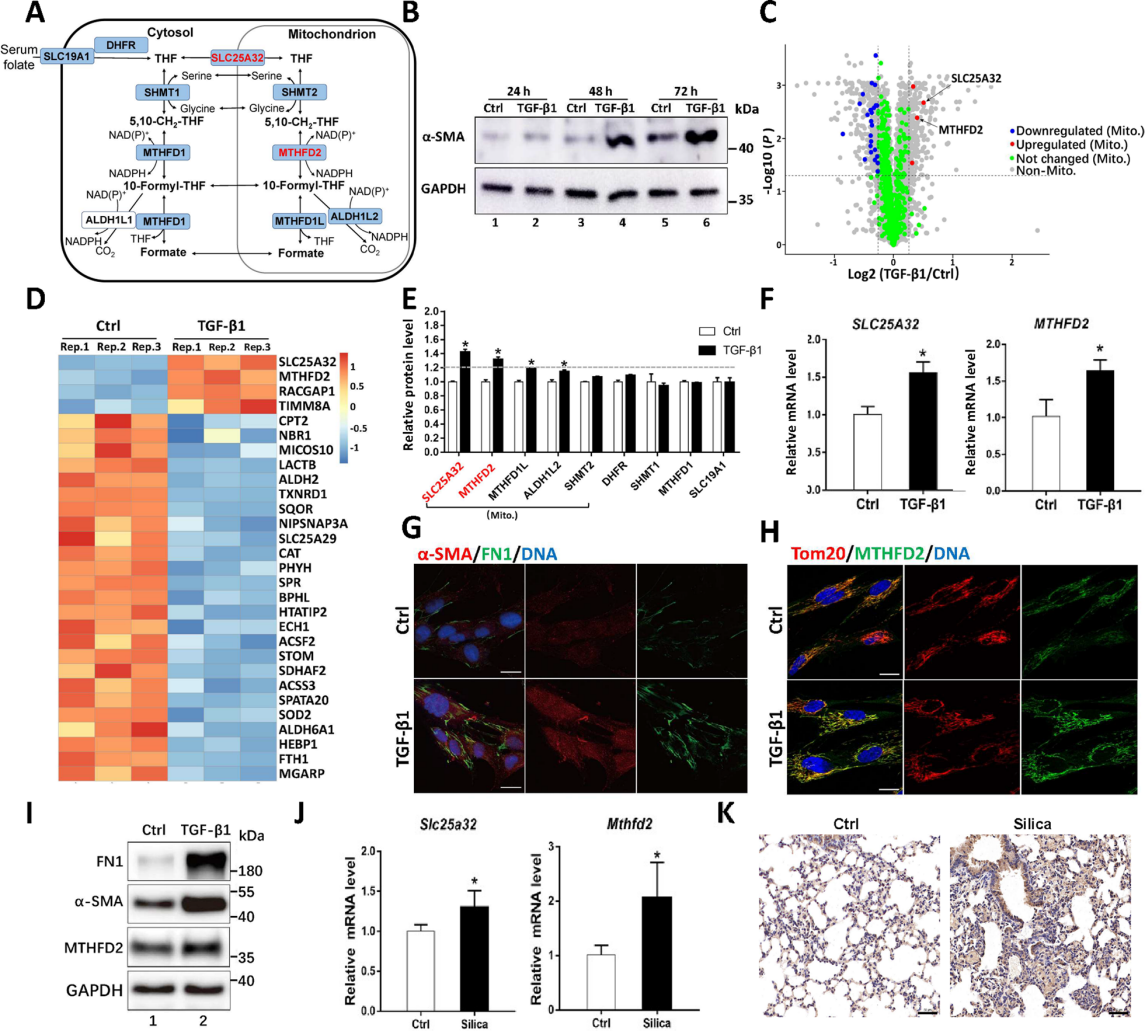
Mitochondrial folate pathway is involved in myofibroblast differentiation and silica-induced pulmonary fibrosis. **A** Schematic diagram of folate metabolism. Proteins with blue frame were detected by mass spectrometry. Proteins highlighted in red were upregulated. **B** Lysates from MRC-5 cells treated with or without TGF-β1 for different time were analyzed by western blotting with indicated antibodies. **C** Volcano plot of proteins quantified by mass spectrometry following TGF-β1 treatment for 48 h. Mitochondrial proteins were highlighted with indicated color. **D** Heatmap of differentially expressed mitochondrial proteins detected by mass spectrometry. **E** Relative abundance of proteins involved in cytosolic and mitochondrial folate pathway detected by mass spectrometry. Proteins highlighted in red were above the upregulation threshold dash line. **F** *SLC25A32* and *MTHFD2* expression levels in MRC-5 with or without TGF-β1 treatment for 48 h were determined by RT-qPCR. Results are expressed as mean ± SD, n = 3, * represents* P* < 0.05. **G**, **H** Immunofluorescence of MRC-5 cells with indicated antibodies following TGF-β treatment for 48 h. Scale bar = 20 μm. **I** Western blotting of MRC-5 cells with indicated antibodies following TGF-β treatment for 48 h. **J** *Slc25a32* and *Mthfd2* expression levels in normal and fibrotic lung tissue of mice were determined by RT-qPCR. Results are expressed as mean ± SD, n = 4, * represents *P* < 0.05. **K** Immunohistochemical staining of MTHFD2 in normal and silica induced fibrotic lung tissue of mice. Scale bar = 50 μm

The regeneration of cytosolic NADPH from NADP^+^ relies primarily on the oxidative pentose phosphate pathway. Recently, folate mediated one-carbon (1C) metabolism has been reported as another important source for NADPH production [28, 29]. Folate metabolism occurs mainly in cytosol and mitochondria (Fig. 1A). After being transported into cell, folic acid is converted via dihydrofolate reductase (DHFR) to its reduced form tetrahydrofolate (THF), which is imported into mitochondrial matrix via mitochondrial folate carrier encoded by SLC25A32 [30, 31]. The THF accepts the 1C unit from serine under the reaction of mitochondrial serine hydroxymethyltransferase 2 (SHMT2) to produce glycine and 5,10-methylene-tetrahydrofolate (5,10-methylene-THF). 5,10-Methylene-THF is subsequently oxidized to 10-formyl-THF while generating NADPH via methylenetetrahydrofolate dehydrogenase 2 (MTHFD2) [32]. The 1C unit of 10-formyl-THF has three possible fates. It can be released as CO_2_ while generating NADPH by 10-formyl-THF dehydrogenase (ALDH1L2); it can be used to formylate mitochondrial methionyl-tRNA to produce *N*-formylmethionine-tRNA (fMet-tRNA) for translation initiation; under the reaction of MTHFD1L, it is metabolized to formate, which can be exported into cytosol for the synthesis of purine and thymidylate as well as for homocysteine remethylation. Folate metabolism plays important roles in development and cancer, but how it regulates myofibroblast differentiation and silica-induced lung fibrosis remains unclear [33, 34].

In this study, by quantitative mass spectrometry, we revealed that proteins involved in mitochondrial folate metabolism were specifically upregulated during myofibroblast formation following TGF-β stimulation. Impairing the expression of mitochondrial folate metabolic protein MTHFD2 or SLC25A32 promoted myofibroblast differentiation, while its overexpression suppressed this process. Moreover, we observed that plasma folate concentration was significantly reduced in patients and mice with silicosis. Folate supplementation elevated the expression of MTHFD2 and SLC25A32, alleviated oxidative stress and effectively suppressed myofibroblast formation and silica-induced pulmonary fibrosis in mice. Together, our study suggests for the first time that mitochondrial folate pathway regulates myofibroblast differentiation and could serve as a potential target for ameliorating silica-induced pulmonary fibrosis.

## Methods

### Cell culture and transfection

Human lung fibroblast MRC-5 (National Collection of Authenticated Cell Cultures, GNHu41, passage 22-36) was cultured in Dulbecco's Modified Eagle Medium (DMEM) (Solarbio, 11995) supplemented with 10% fetal bovine serum (FBS) (Hyclone, SV30087.03) and 1% sodium pyruvate (Solarbio, P8380) at 37 °C with 5% CO_2_. MCR-5 was stimulated with 5 ng/mL TGF-β1 (PEPROTECH, 100-21) for 48 h to induce fibroblast transdifferentiation with or without 50 μM 5-methyltetrahydrofolic acid (5-mTHF, MedChemExpress, 31690-09-2) pretreatment for 12 h unless otherwise specified. The siRNA duplexes for MTHFD2, SLC25A32 and negative control were obtained from GenePharma (Additional file 7: Table S1) and were transfected at the final concentration 15 nM by Lipofectamine RNAiMAX (Thermo Fisher, 13,778,030) according to the manufacturer’s instructions. Expression plasmids for MTHFD2, SLC25A32 and negative control were obtained from Genechem and verified by sequencing. Plasmids of 4 μg per well of 6-well plate were transfected by Lipofectamine 3000 (Thermo Fisher, L3000008) according to the manufacturer’s instructions.

### Mouse model for silicosis

All mice experiments were approved by Zhengzhou University Animal Policy and Welfare Committee and were performed according to relevant guidelines. C57BL/6N male mice (5–6 weeks after birth) were purchased from Charles River Laboratories. Mice were acclimated to the new laboratory environment for 1–2 weeks and then were randomly assigned to receive a single dose intratracheal instillation with 100 μL 50 mg/mL silica suspension (Sigma-Aldrich, S5639) or saline as control for four weeks before sacrifice. For the folic acid treatment, mice were supplemented with 20 μg/mL folic acid (BBI Life Science, A610466-0025) or dimethyl sulfoxide (DMSO) as control in drinking water one week before silica instillation [35].

### Analysis of plasma folate concentration

Fasting peripheral blood samples were collected from male silicosis patients (n = 18, average age: 69.29 ± 8.79) and healthy control (n = 29, average age: 69.62 ± 8.88). Informed consents were obtained from all participants. The study was approved by the Human Research Ethics Committee of Zhengzhou University. The fasting peripheral blood stood at room temperature for 30 min followed by centrifugation at 3000 rpm for 10 min, and the upper plasma was carefully collected for folate concentration analysis by ELISA (Elabscience, E-EL-0009c) according to the manufacturer's instructions. Folate concentration in mouse plasma was measured in the same way.

### Histologic analysis of lung sections

Mice lung tissue was fixed in 4% paraformaldehyde for more than 24 h and embedded in paraffin. The paraffin-embedded samples were frozen at – 20 °C and cut into slices with a thickness of 4 µm, then dried in the oven at 60 °C and stored at room temperature. Tissue sections were stained with H&E, Masson’s trichrome or antibodies against MTHFD2 (Proteintech, 12270-1-AP), FN1 (Servivebio, GB114491), COL1A1(Servivebio, GB11022-3) or α-SMA (Servivebio, GB111364). Images were obtained using a Pannoramic MIDI slice digital scanner.

### Western blotting

Proteins from MRC-5 cells were extracted by RIPA buffer (50 mM Tris pH 7.4, 150 mM NaCl, 1% Triton X-100, 1% sodium deoxycholate, 0.1% SDS) supplemented with 1 mM PMSF (Dingguo, WB-0072) and protein concentration was measured by BCA protein assay kit (BOSTER, AR0146). Protein samples were separated by SDS-PAGE, transferred onto polyvinylidene fluoride (PVDF) membranes (Millipore, ISEQ00010) and detected with primary antibodies against GAPDH (CST, D16H11), α-SMA (Servivebio, GB111364), FN1 (Proteintech, 15613-1-AP), COL1A1 (Abcam, ab260043) or MTHFD2 (Proteintech, 12270-1-AP). Membranes were subsequently incubated with horseradish peroxidase-conjugated secondary antibodies (CST, 7074) and developed with ECL luminescence reagent (Absin, abs920).

### Immunofluorescence

Cells were fixed with 4% paraformaldehyde, permeabilized with 0.1% Triton X-100, incubated with primary antibodies against FN1 (Proteintech, 15613-1-AP), α-SMA (Servicebio, GB111364), Tom20 (SCBT, sc-17764), MTHFD2 (Proteintech, 12270-1-AP) or anti-DNA (Progen, 61014) and subsequently detected by the fluorophore-labeled secondary antibodies (Invitrogen, A11034 or A21236). To detect mitochondrial membrane potential, cells were incubated with 100 nM MitoTracker-Red (Invitrogen, M7512) for 30 min before fixation. Nuclear DNA was stained by Hoechst 33258 (Sigma-Aldrich, 14530).

### Real-time quantitative polymerase chain reaction (RT-qPCR)

RNAiso Plus reagent (Takara, 9109) was used to extract total RNA from cultured cells and lung tissue. DNase (Takara, RR047A) was added to eliminate genomic DNA contamination, and cDNA was synthesized using the reverse transcription kit (Takara, RR047A). The primers for polymerase chain reaction (PCR) were listed in Additional file 7: Table S1. Diluted cDNA templates were then used for RT-qPCR analysis using a TB Green® Premix Ex Taq™ II Kit (Takara, RR820A) for target genes. The expression levels of the target genes were normalized to GAPDH and expressed as fold changes using the 2^−△△t^ method.

### Quantitative mass spectrometry

Cultured cells (triplicates for both control and TGF-β treated cells) were collected and washed with PBS. Proteins were extracted by SDT (4% (w/v) SDS, 100 mM Tris/HCl pH 7.6, 0.1 M DTT) lysis buffer and taken for trypsin enzymolysis by filter aided proteome preparation (FASP) method [36]. The peptides were quantified by OD_280_ and labelled with TMT reagents (Thermo Fisher, 90064CH). Labeled peptides were graded using the High pH Reversed-Phase Peptide Fractionation Kit (Thermo Fisher, 84868), and then were loaded in buffer A (0.1% (v/v) formic acid) at a flow rate of 300 nL/min. Separated samples were analyzed by mass spectrometry using Q-Exactive mass spectrometer. Raw data were identified and quantitatively analyzed using software Mascot 2.2 and Proteome Discoverer 1.4.

### Measurement of ROS

Intracellular total ROS was analyzed by DCFH-DA (Beyotime, S0033S) and mitochondrial superoxide was detected by MitoSOX Red (Invitrogen, M36008) according to the manufacturer’s instructions. ROS-dependent dichlorofluorescein fluorescence was monitored using a BD Accuri C6 flow cytometry (BD Biosciences) at an emission wavelength of 525 nm and an excitation wavelength of 488 nm. Mitochondrial ROS fluorescence was monitored at an emission wavelength of 580 nm and an excitation wavelength of 510 nm. The analysis of H_2_O_2_ (Beyotime, S0101S) and MDA (Nanjing Jiancheng Bioengineering Institute, A003-4-1) in cells and lung tissue was performed according to the manufacturer’s instructions.

### Statistical analysis

Statistical analyses were performed using SPSS 21.0. Significance between two groups was determined by the two tailed Student’s* t*-test. Significance for pairwise comparison among more than two groups was determined by one-way analysis of variance (ANOVA) with LSD post hoc test. Statistical significance was set at *P* < 0.05. For multiple comparisons issue, we adjusted *P-values* based on the Benjamini and Hochberg (BH) method and ensured that the false discovery rate (FDR) < 0.05 [37]. Data were expressed as mean ± standard deviation (SD).

## Results

### Mitochondrial folate pathway is involved in myofibroblast differentiation and silica-induced pulmonary fibrosis

To induce myofibroblast differentiation, human lung fibroblasts were stimulated with TGF-β for different time period. The steady levels of marker protein α-SMA was significantly increased after 48 h treatment (Fig. 1B). To systematically explore the regulatory mechanisms of myofibroblast formation in an unbiassed way, lysates from cells treated with TGF-β for 48 h and the control group were subjected to tandem mass tags (TMT)-based quantitative proteomics. A total of 5586 different proteins were identified, of which 8.7% were differentially expressed (fold change above 1.2 or below its reciprocal, FDR-adjusted *p* value < 0.05) (Additional file 8: Table S2). Among them, 217 and 270 different proteins were upregulated or downregulated after TGF-β stimulation, respectively (Additional file 1: Fig. S1A). As expected, proteins involved in TGF-β signaling, extracellular matrix remodeling and wound repairing were enriched [38] (Additional file 1: Fig. S1B-S1F).

To gain insights about how mitochondria may regulate myofibroblast differentiation, we first combined the genes from MitoCarta3.0 database [39] (1136 human genes) with a recently published human mitochondrial high-confidence proteome MitoCoP [40] (1134 genes) to generate a mitochondrial inventory with 1310 genes (Additional file 1: Fig. S1G, Additional file 8: Table S3). According to this inventory, 680 different mitochondrial proteins (Additional file 1: Fig. S1H, Additional file 8: Table S4) were identified in our quantitative proteomic analysis with 29 differentially expressed (4 upregulated, 25 downregulated) (Fig. 1C, D). Interestingly, two of the upregulated genes, SLC25A32 and MTHFD2, encode key proteins involved in mitochondrial folate metabolism. Further manual inspection of the proteomic data discovered that another two key proteins (MTHFD1L and ALDH1L2) of mitochondrial folate metabolic pathway were significantly upregulated, though the fold change was below the threshold (Fig. 1E). Of note, proteins involved in cytosolic folate metabolism seemed not to be affected following TGF-β stimulation (Fig. 1E).

To verify whether mitochondrial folate pathway may participate in myofibroblast differentiation, we focused on analyzing SLC25A32 and MTHFD2 and found that their expression levels were indeed increased after TGF-β treatment (Fig. 1F–I). To gain further insights into the involvement of mitochondrial folate pathway in silicosis, mice were intratracheally instilled with silica suspension for four weeks to induce pulmonary fibrosis, and the expression levels of *Slc25a32* and *Mthfd2* in fibrotic lung tissue were indeed elevated comparing to the control (Fig. 1J, K). Meanwhile, we observed that the plasma folate concentration of mice treated with silica (6.51 ± 2.50 ng/mL) was significantly lower than that of the control group (11.19 ± 4.20 ng/mL) (Additional file 1: Fig. S1I). Similarly, the mean plasma folate concentration of silicosis patients (5.69 ± 1.66 ng/mL) was significantly lower than the healthy control (6.72 ± 1.16 ng/mL) (Additional file 1: Fig. S1J, K).

Together, these findings suggest that folate metabolism may be dysregulated in silicosis and mitochondrial folate pathway may be involved in myofibroblast differentiation and silica-induced pulmonary fibrosis.

### MTHFD2 and SLC25A32 negatively regulate TGF-β induced myofibroblast differentiation

**Fig. 2:**
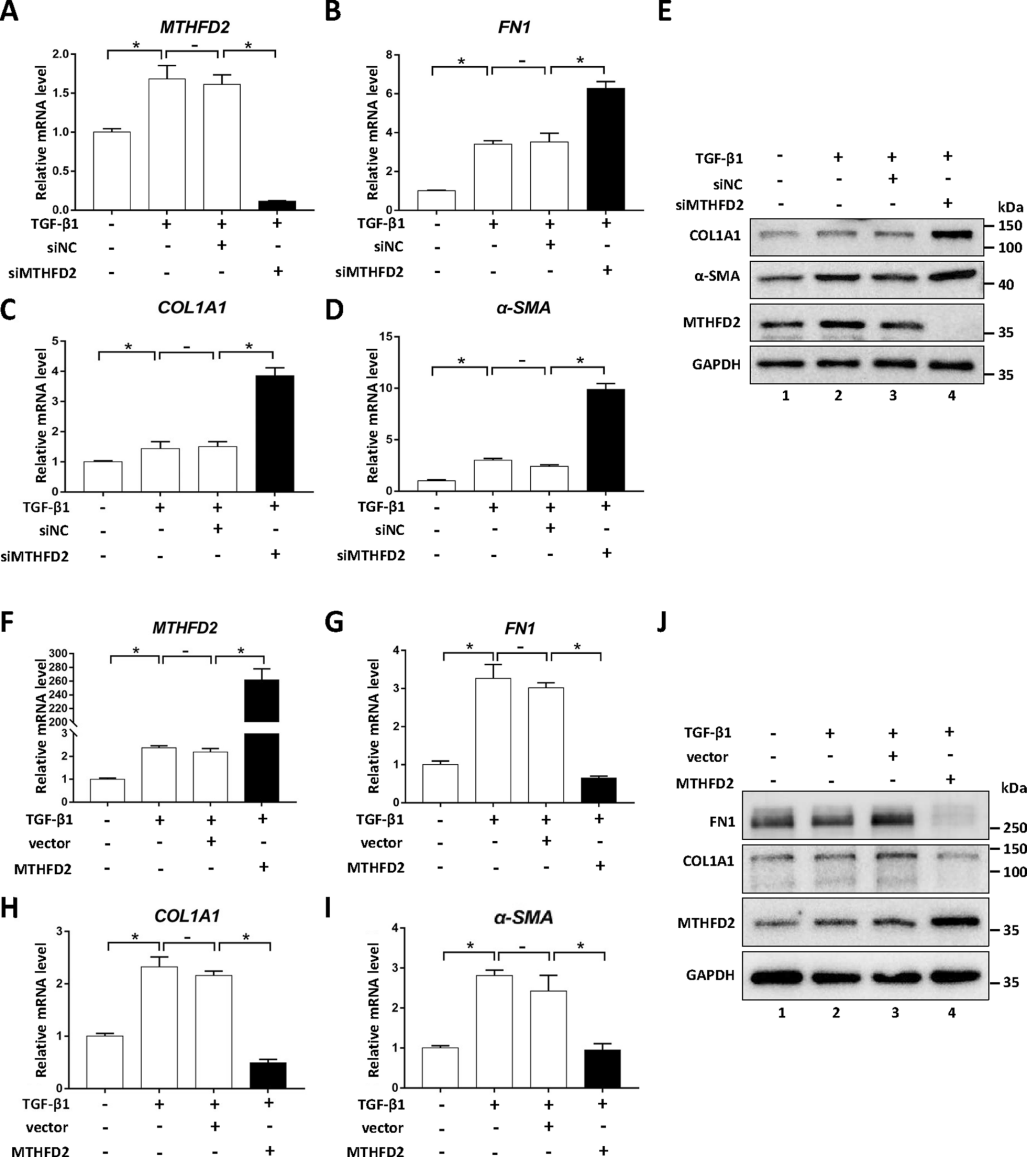
MTHFD2 negatively regulates TGF-β induced myofibroblast differentiation. **A**–**D** The mRNA levels of *MTHFD2* and myofibroblast markers (*FN1*, *COL1A1*, *α-SMA*) in cells treated with siRNA and TGF-β1 as indicated for 48 h were determined by RT-qPCR. Results are expressed as mean ± SD, n = 3, * represents *P* < 0.05. **E** Lysates from cells treated with siRNA and TGF-β1 as indicated for 48 h were analyzed by western blotting with indicated antibodies. **F**–**I** The mRNA levels of *MTHFD2* and myofibroblast markers (*FN1*, *COL1A1*, *α-SMA*) in cells transfected with indicated plasmids were determined by RT-qPCR following TGF-β1 treatment for 48 h. Results are expressed as mean ± SD, n = 3, * represents *P* < 0.05. **J** Lysates from cells transfected with indicated plasmids were analyzed by western blotting with various antibodies following TGF-β1 treatment for 48 h

To analyze how mitochondrial folate pathway may regulate myofibroblast differentiation, we first suppressed the gene expression of MTHFD2 by siRNA knockdown. The downregulation of MTHFD2 further stimulated the expression of fibrotic marker genes FN1, COL1A1 and α-SMA following TGF-β treatment, suggesting that mitochondrial folate pathway may negatively regulate TGF-β induced myofibroblast differentiation (Fig. 2A–E). To upregulate the expression of MTHFD2, plasmid encoding the gene was transfected, and cells were analyzed after 48 h following TGF-β treatment. The enhanced expression of MTHFD2 significantly inhibited the expression of fibrotic marker genes (Fig. 2F–J). Moreover, regulating the expression of SLC25A32 by siRNA knockdown or transient overexpression leads to similar observations (Additional file 2: Fig. S2A–D). Together, these data indicated that the key proteins of mitochondrial folate pathway, MTHFD2 and SLC25A32, could negatively regulate TGF-β induced myofibroblast differentiation.

### Folate supplementation suppresses TGF-β induced oxidative stress during myofibroblast differentiation

**Fig. 3:**
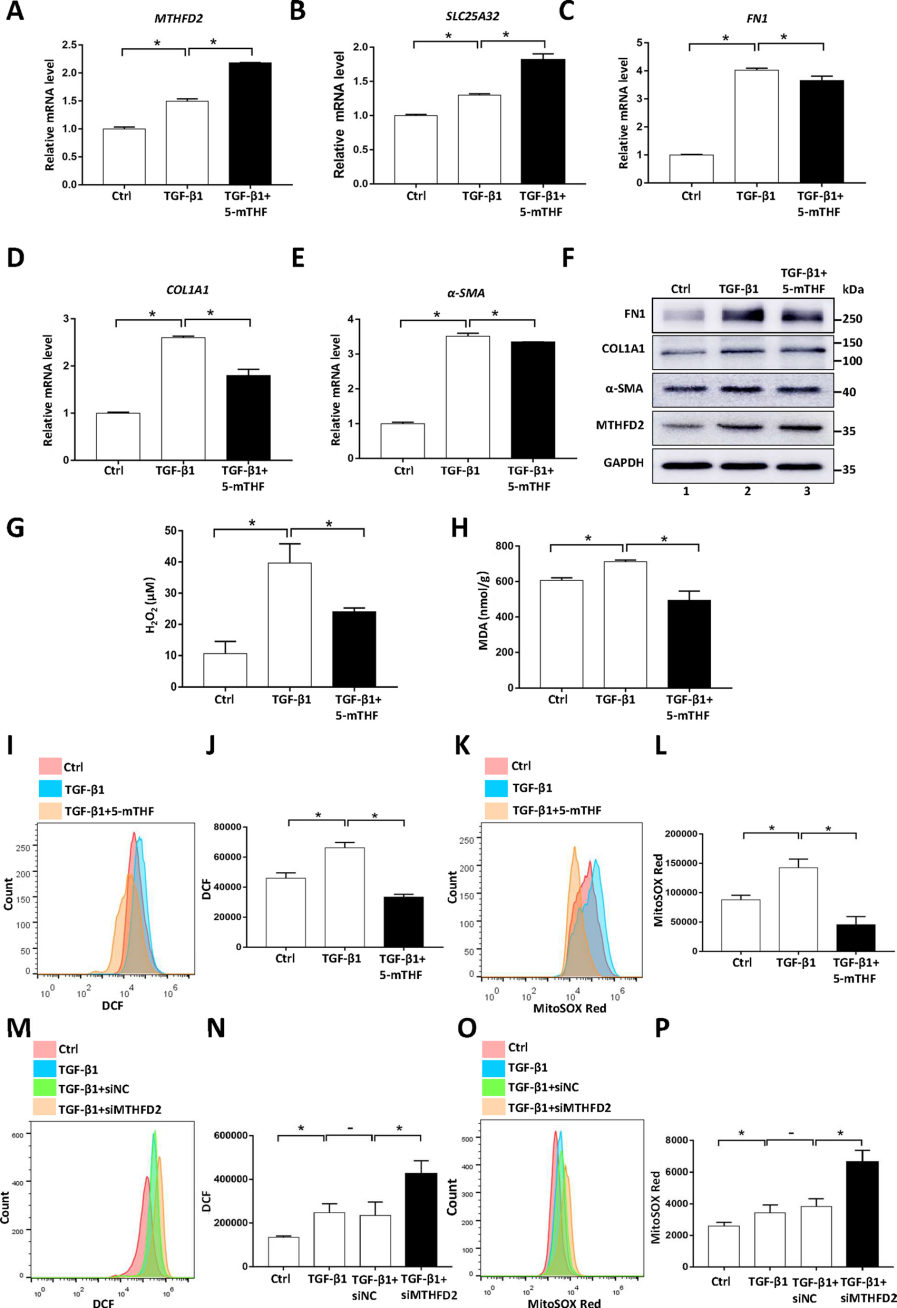
Folate supplementation suppresses TGF-β induced oxidative stress during myofibroblast differentiation. **A**–**E** Gene expression levels in cells treated with 5-mTHF and TGF-β1 as indicated were determined by RT-qPCR. Results are expressed as mean ± SD, n = 3, * represents *P* < 0.05. **F** Lysates from cells treated with 5-mTHF and TGF-β1 as indicated were analyzed by western blotting. **G**, **H** Concentration analysis of H_2_O_2_ and MDA in cells treated with 5-mTHF and TGF-β1 as indicated. Results are expressed as mean ± SD, n = 3, * represents *P* < 0.05. **I**–**L** Flow cytometry analysis of total intracellular ROS (DCF) and mitochondrial ROS (MitoSOX Red) in cells treated with 5-mTHF and TGF-β1 as indicated. Results are expressed as mean ± SD, n = 3, * represents *P* < 0.05. **M**–**P** Flow cytometry analysis of total intracellular ROS (DCF) and mitochondrial ROS (MitoSOX Red) in cells treated with siRNA and TGF-β1 as indicated. Results are expressed as mean ± SD, n = 3, * represents *P* < 0.05

Next, we asked the question whether folate supplementation could impair myofibroblast differentiation. As a predominant form of circulating folate, 5-mTHF could be readily utilized by cultured cells [41, 42]. As expected, supplementing 5-mTHF in concomitant with TGF-β further elevated the expression of MTHFD2 and SLC25A32 (Fig. 3A, 3B and 3F). Moreover, the TGF-β stimulated expression of fibrotic marker genes was reduced to different extent by folate supplementation (Fig. 3C–F). Folate supplementation did not cause obvious morphological changes of cells (Additional file 6: Fig. S6). Previous studies reported that oxidative stress plays important roles in promoting myofibroblast differentiation [22–25]. Accordingly, we observed that the major mitochondrial ROS scavenger protein, superoxide dismutase 2 (SOD2), was downregulated following TGF-β stimulation (Fig. 1D, Additional file 3: Fig. S3A, S3B). Moreover, TGF-β treatment increased the cellular level of H_2_O_2_ and malondialdehyde (MDA) as well as the ROS dependent signal intensities of DCF and MitoSOX Red, indicating the presence of oxidative stress (Fig. 3G–L). Importantly, supplementing folate significantly suppressed the oxidative stress induced by TGF-β (Fig. 3G–L). To verify whether mitochondrial folate pathway is important for limiting the oxidative stress, MTHFD2 or SLC25A32 was downregulated by siRNA transfection, and we observed that the levels of total ROS and mitochondrial ROS were both further upregulated following TGF-β treatment (Fig. 3M–P, Additional file 3: Fig. S3C–S3F). Taken together, these data demonstrated that supplementing folate could suppress the TGF-β induced oxidative stress via mitochondrial folate pathway.

### Folate supplementation effectively reduces silica-induced pulmonary fibrosis and oxidative stress

**Fig. 4:**
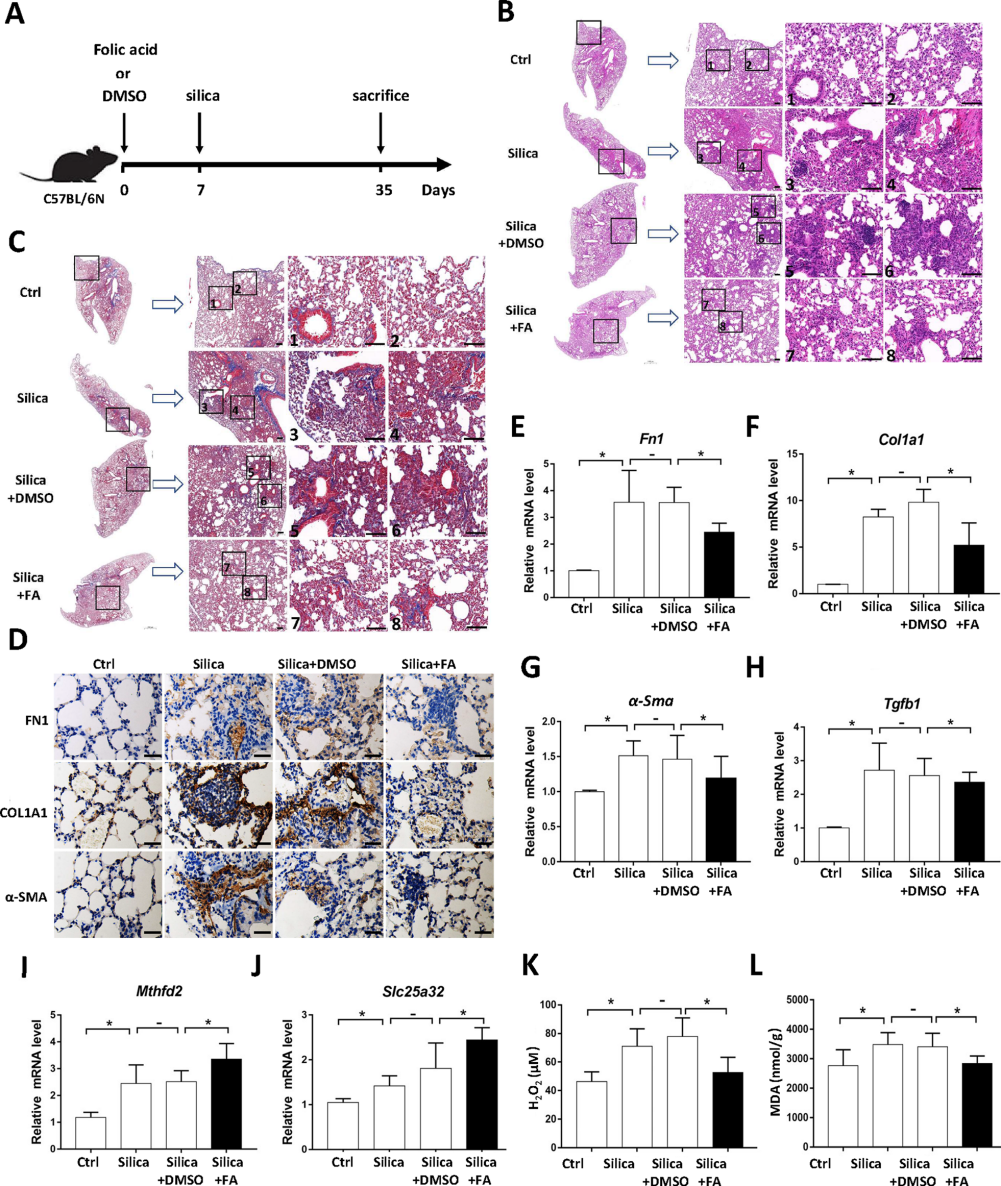
Folate supplementation effectively reduces silica-induced pulmonary fibrosis and oxidative stress. **A** Schematic diagram of folic acid (FA) supplementation in silica-induced pulmonary fibrosis mouse model. **B**, **C** Paraffin-embedded sections of lung tissue was stained with H&E and Masson staining, respectively. The areas marked with numbers are further enlarged. Scale bar = 100 μm. **D** Paraffin-embedded sections of lung tissue were analyzed by immunohistochemical staining with indicated antibodies. Scale bar = 100 μm. **E**–**J** Gene expression levels of different lung tissues were determined by RT-qPCR. Results are expressed as mean ± SD, n = 4 mice/group, * represents *P* < 0.05. **K**, **L** Concentration analysis of H_2_O_2_ and MDA of different lung tissues. Results are expressed as mean ± SD, n = 4 mice/group, * represents *P* < 0.05

To assess whether folate has therapeutic potential in silica-induced pulmonary fibrosis, we supplemented folic acid in drinking water for mice one week before intratracheal instillation of silica suspension (Fig. 4A). No significant difference in body weight was observed among different groups (Additional file 4: Fig. S4A). However, the lung weight and lung/body weight ratio were significantly increased in mice treated with silica (Additional file 4: Fig. S4B, S4C). Nodules of varying size and number were observed on the surface of lungs in silica group and silica + DMSO group (Additional file 4: Fig. S4D). Folate supplementation alleviated the occurrence of pulmonary nodules, but did not significantly affect the weights of body and lung, neither the lung/body weight ratio (Additional file 4: Fig. S4A–S4D).

Subsequently, paraffin-embedded sections of lung tissue were stained with H&E and Masson staining. H&E staining showed that silica caused alveolar destruction, alveolar wall thickening, inflammatory cell aggregation and fibrous nodule formation in lung tissue, while the silica caused lung tissue damage was alleviated in the folate supplementation group (Fig. 4B). Masson staining showed that silica led to increased collagen deposition at the lesion site of lung tissue, indicating the occurrence of pulmonary fibrosis, while the collagen deposition was reduced in mice supplemented with folate (Fig. 4C). Moreover, folate supplementation mitigated the silica-induced expression of fibrotic markers in lung tissue (Fig. 4D–H), enhanced the expression of *Mthfd2* and *Slc25a32* (Fig. 4I–J), and inhibited the silica-induced oxidative stress in lung tissue of mice (Fig. 4K–L). Collectively, these data demonstrated that folate supplementation effectively reduced silica-induced pulmonary fibrosis and oxidative stress.

## Discussion

Silicosis is a progressive and irreversible disease, in which the accumulated SiO_2_ particles in lung cause persistent inflammation and tissue damage, eventually leading to respiratory failure and death [43]. It is widely accepted that fibroblast activation is a key event in lung fibrogenesis and excessive amounts of ROS can promote myofibroblast differentiation and pulmonary fibrosis [22–25]. Therefore, to investigate the ways limiting ROS generation may provide new therapeutic opportunities for silicosis. Recently, folate mediated 1C metabolism has been reported as an important source for the production of NADPH, which is a key molecule to provide cellular reducing power against oxidative stress [28, 29].

In this study, proteins involved in mitochondrial folate metabolism were specifically upregulated during myofibroblast formation in our unbiased analysis by quantitative mass spectrometry. Interestingly, glucose-6-phosphate dehydrogenase (G6PD) and isocitrate dehydrogenase 1 (IDH1), enzymes crucial for cytosolic NADPH production, were significantly downregulated (Additional file 5: Fig. S5A). It is conceivable that the enhanced expression of mitochondrial folate pathway may be poised to compensate the cellular NADPH production, since NADPH production by oxidative pentose-phosphate pathway has been reported to be interconnected to folate metabolism [44]. Further analysis indicated that the expression of MTHFD2 and SLC25A32 negatively regulated myofibroblast differentiation following TGF-β stimulation. Interestingly, the plasma folate concentration was observed to be significantly lower in patients and mice with silicosis. Importantly, folate supplementation elevated the expression of MTHFD2 and SLC25A32, suppressed TGF-β induced oxidative stress and effectively mitigated myofibroblast formation and silica-induced pulmonary fibrosis in mice.

Mitochondrial DNA (mtDNA) replication defects have been reported to remodel 1C metabolism, leading to substantially increased expression of MTHFD2 [44]. In our study, TGF-β enhanced the expression level of MTHFD2 mildly, and the levels of proteins involved in mitochondrial central dogma were not observed to be differentially regulated (Additional file 8: Table S5). Indeed, qPCR and immunofluorescence did not reveal any significant difference of mtDNA level in cells treated with TGF-β comparing to the control (Additional file 5: Fig. S5B–S5G), indicating that TGF-β stimulated mitochondrial folate pathway was not associated with detectable defects of mtDNA replication during myofibroblast differentiation. Of note, a previous report observed increased mtDNA content in TGF-β induced myofibroblast differentiation, which could be due to different biological material or/and experimental conditions used [16].

Folate is a crucial cofactor to transfer 1C unit for purine and thymidine biosynthesis and methylation reactions, thus is important for neural tube development and cancer cell biology [33, 34]. Folate uptake can be mediated via reduced folate carrier (SLC19A1), proton-coupled folate transporter (SLC46A1) or folate receptors (FOLR1/2/3) [45]. Though the latter two types were not detected in our quantitative mass spectrometry, the expression level of SLC19A1 was not significantly affected by TGF-β treatment, along with the cytosolic folate metabolic proteins. Interestingly, folate receptor β (FRβ, encoded by FOLR2) is highly expressed on lung macrophages from patients with idiopathic pulmonary fibrosis and from bleomycin treated mice, and has been exploited to selectively target therapeutic agents to suppress the production of profibrotic cytokines by activated macrophages [46, 47]. However, the physiological relevance of upregulated FRβ on activated pulmonary macrophages is unclear. Certainly, we could not exclude the effects of folate supplementation beyond redox homeostasis and myofibroblast differentiation. The regulatory mechanisms of folate metabolism on macrophage activities in pulmonary fibrosis await further studies.

## Conclusion

In summary, our study indicated that mitochondrial folate pathway may serve as a key switch to regulate oxidative stress during myofibroblast differentiation, and folate supplementation could effectively alleviate oxidative stress and suppress myofibroblast differentiation in silica-induced pulmonary fibrosis. Folate is worthy of further research for the treatment of silica-induced pulmonary fibrosis.

## Supplementary Information

**Additional file 1: Figure S1.** Enrichment analysis of differentially expressed proteins identified by mass spectrometry and plasma folate analysis, related to Fig. 1.

**Additional file 2: Figure S2.** SLC25A32 negatively regulates TGF-β induced myofibroblast differentiation, related to Fig. 2.

**Additional file 3: Figure S3.** Suppressing SLC25A32 promotes TGF-β1 induced oxidative stress during myofibroblast differentiation, related to Fig. 3.

**Additional file 4: Figure S4.** The effect of folate supplementation on weight and nodules in silica-treated mice, related to Fig. 4.

**Additional file 5: Figure S5.** The regulation of cytosolic NADPH production pathway and mtDNA level during TGF-β induced myofibroblast differentiation.

**Additional file 6: Figure S6.** The effects of folate supplementation during myofibroblast differentiation.

**Additional file 7: Table S1.** siRNAs and qPCR primers used in this study.

**Additional file 8: Table S2.** Proteins identified by quantitative mass spectrometry. Table S3 Mitochondrial inventory combining MitoCarta3.0 and MitoCoP. Table S4 Mitochondrial proteins identified by quantitative mass spectrometry. Table S5 Proteins involved in mitochondrial central dogma identified by quantitative mass spectrometry.

## Data Availability

The data presented in this study are available on request from the corresponding authors.

## References

[1] Wagner GR (1997). Asbestosis and silicosis. Lancet.

[2] Otsuki T, Maeda M, Murakami S (2007). Immunological effects of silica and asbestos. Cell Mol Immunol.

[3] Mossman BT, Churg A (1998). Mechanisms in the pathogenesis of asbestosis and silicosis. Am J Respir Crit Care Med.

[4] Noble PW, Barkauskas CE, Jiang D (2012). Pulmonary fibrosis: patterns and perpetrators. J Clin Invest.

[5] Kis K, Liu X, Hagood JS (2011). Myofibroblast differentiation and survival in fibrotic disease. Expert Rev Mol Med.

[6] Tomasek JJ, Gabbiani G, Hinz B (2002). Myofibroblasts and mechano-regulation of connective tissue remodelling. Nat Rev Mol Cell Biol.

[7] Hardie WD, Glasser SW, Hagood JS (2009). Emerging concepts in the pathogenesis of lung fibrosis. Am J Pathol.

[8] Thannickal VJ, Toews GB, White ES (2004). Mechanisms of pulmonary fibrosis. Annu Rev Med.

[9] Hinz B, Lagares D (2020). Evasion of apoptosis by myofibroblasts: a hallmark of fibrotic diseases. Nat Rev Rheumatol.

[10] Hinz B, Phan SH, Thannickal VJ (2012). Recent developments in myofibroblast biology: paradigms for connective tissue remodeling. Am J Pathol.

[11] Hinz B, Phan SH, Thannickal VJ (2007). The myofibroblast: one function, multiple origins. Am J Pathol.

[12] Hinz B (2012). Mechanical aspects of lung fibrosis: a spotlight on the myofibroblast. Proc Am Thorac Soc.

[13] Liu G, Philp AM, Corte T (2021). Therapeutic targets in lung tissue remodelling and fibrosis. Pharmacol Ther.

[14] Rangarajan S, Bone NB, Zmijewska AA (2018). Metformin reverses established lung fibrosis in a bleomycin model. Nat Med.

[15] Xie N, Tan Z, Banerjee S (2015). Glycolytic reprogramming in myofibroblast differentiation and lung fibrosis. Am J Respir Crit Care Med.

[16] Bernard K, Logsdon NJ, Ravi S (2015). Metabolic reprogramming is required for myofibroblast contractility and differentiation. J Biol Chem.

[17] Negmadjanov U, Godic Z, Rizvi F (2015). TGF-beta1-mediated differentiation of fibroblasts is associated with increased mitochondrial content and cellular respiration. PLoS ONE.

[18] Murphy MP (2009). How mitochondria produce reactive oxygen species. Biochem J.

[19] Bratic I, Trifunovic A (2010). Mitochondrial energy metabolism and ageing. Biochim Biophys Acta.

[20] Gonzalez C, Sanz-Alfayate G, Agapito MT (2002). Significance of ROS in oxygen sensing in cell systems with sensitivity to physiological hypoxia. Respir Physiol Neurobiol.

[21] Baran CP, Zeigler MM, Tridandapani S (2004). The role of ROS and RNS in regulating life and death of blood monocytes. Curr Pharm Des.

[22] Alili L, Sack M, Puschmann K (2014). Fibroblast-to-myofibroblast switch is mediated by NAD(P)H oxidase generated reactive oxygen species. Biosci Rep.

[23] Jain M, Rivera S, Monclus EA (2013). Mitochondrial reactive oxygen species regulate transforming growth factor-beta signaling. J Biol Chem.

[24] Toullec A, Gerald D, Despouy G (2010). Oxidative stress promotes myofibroblast differentiation and tumour spreading. EMBO Mol Med.

[25] Cheresh P, Kim SJ, Tulasiram S (2013). Oxidative stress and pulmonary fibrosis. Biochim Biophys Acta.

[26] Handy DE, Loscalzo J (2012). Redox regulation of mitochondrial function. Antioxid Redox Signal.

[27] Kaludercic N, Deshwal S, Di Lisa F (2014). Reactive oxygen species and redox compartmentalization. Front Physiol.

[28] Fan J, Ye J, Kamphorst JJ (2014). Quantitative flux analysis reveals folate-dependent NADPH production. Nature.

[29] Lewis CA, Parker SJ, Fiske BP (2014). Tracing compartmentalized NADPH metabolism in the cytosol and mitochondria of mammalian cells. Mol Cell.

[30] Kim J, Lei Y, Guo J (2018). Formate rescues neural tube defects caused by mutations in Slc25a32. Proc Natl Acad Sci U S A.

[31] McCarthy EA, Titus SA, Taylor SM (2004). A mutation inactivating the mitochondrial inner membrane folate transporter creates a glycine requirement for survival of chinese hamster cells. J Biol Chem.

[32] Tibbetts AS, Appling DR (2010). Compartmentalization of mammalian folate-mediated one-carbon metabolism. Annu Rev Nutr.

[33] Qu Y, Hao C, Zhai R (2020). Folate and macrophage folate receptor-beta in idiopathic pulmonary fibrosis disease: the potential therapeutic target?. Biomed Pharmacother.

[34] Ducker GS, Rabinowitz JD (2017). One-carbon metabolism in health and disease. Cell Metab.

[35] Li W, Tang R, Ouyang S (2017). Folic acid prevents cardiac dysfunction and reduces myocardial fibrosis in a mouse model of high-fat diet-induced obesity. Nutr Metab (Lond).

[36] Wisniewski JR, Zougman A, Nagaraj N (2009). Universal sample preparation method for proteome analysis. Nat Methods.

[37] Benjamini Y, Hochberg Y (1995). Controlling the false discovery rate: a practical and powerful approach to multiple testing. J Roy Stat Soc B.

[38] Zhou Y, Zhou B, Pache L (2019). Metascape provides a biologist-oriented resource for the analysis of systems-level datasets. Nat Commun.

[39] Rath S, Sharma R, Gupta R (2021). MitoCarta3.0: an updated mitochondrial proteome now with sub-organelle localization and pathway annotations. Nucleic Acids Res.

[40] Morgenstern M, Peikert CD, Lubbert P (2021). Quantitative high-confidence human mitochondrial proteome and its dynamics in cellular context. Cell Metab.

[41] Kawami M, Honda N, Hara T (2019). Investigation on inhibitory effect of folic acid on methotrexate-induced epithelial-mesenchymal transition focusing on dihydrofolate reductase. Drug Metab Pharmacokinet.

[42] Baggott JE, Tamura T (1999). Bioactivity of orally administered unnatural isomers, [6R]-5-formyltetrahydrofolate and [6S]-5,10-methenyltetrahydrofolate, in humans. Biochim Biophys Acta.

[43] Leung CC, Yu IT, Chen W (2012). Silicosis. Lancet.

[44] Chen L, Zhang Z, Hoshino A (2019). NADPH production by the oxidative pentose-phosphate pathway supports folate metabolism. Nat Metab.

[45] Low PS, Kularatne SA (2009). Folate-targeted therapeutic and imaging agents for cancer. Curr Opin Chem Biol.

[46] Zhang F, Ayaub EA, Wang B (2020). Reprogramming of profibrotic macrophages for treatment of bleomycin-induced pulmonary fibrosis. EMBO Mol Med.

[47] Nagai T, Tanaka M, Hasui K (2010). Effect of an immunotoxin to folate receptor beta on bleomycin-induced experimental pulmonary fibrosis. Clin Exp Immunol.

