# Supplementary material for: Mitochondrial folate pathway regulates myofibroblast differentiation and silica-induced pulmonary fibrosis
Source: J Transl Med. 2023 Jun 6;21:365. doi: 10.1186/s12967-023-04241-0 (PMC10245413; doi:10.1186/s12967-023-04241-0)
Supplement: Supplementary file 1 — Additional file 1: Figure S1. Enrichment analysis of differentially expressed proteins identified by mass spectrometry and plasma folate analysis, related to Fig. 1. [file 12967_2023_4241_MOESM1_ESM.docx]

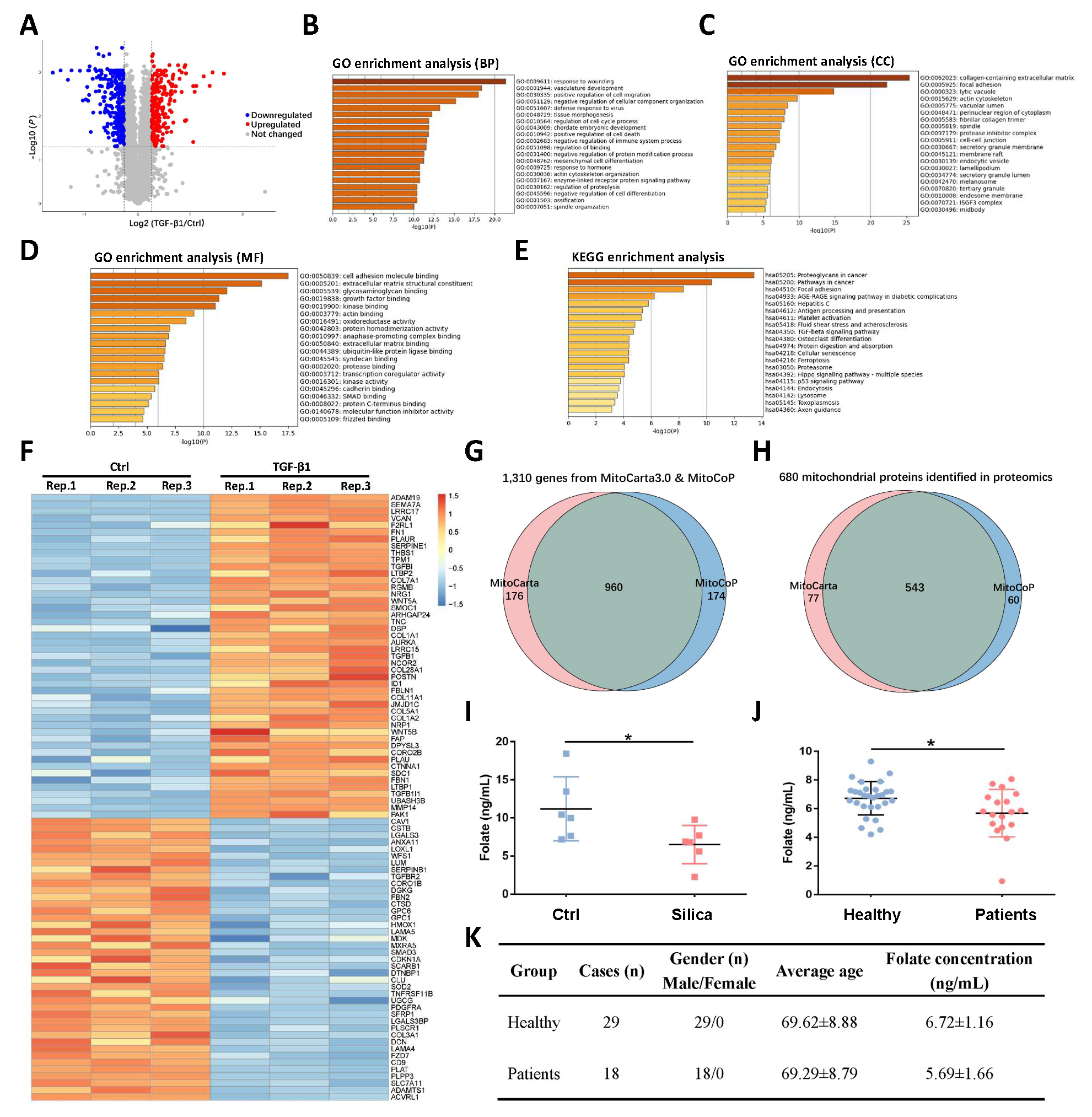


**Figure S1 Enrichment analysis of differentially expressed proteins identified by mass spectrometry and plasma folate analysis, related to Figure 1.**

(A) Volcano plot of proteins quantified by mass spectrometry following TGF-β1 treatment for 48 h. Upregulated and downregulated proteins were colored in red and blue, respectively.

(B)-(E) GO enrichment analysis (BP, CC and MF) and KEGG enrichment analysis of differentially expressed proteins related to (A).

(F) Heatmap of differentially expressed proteins involved in TGF-β signaling, extracellular matrix remodeling and wound repairing.

(G) The Venn diagram of a mitochondrial inventory with 1,310 genes from MitoCarta3.0 and MitoCoP databases.

(H) The Venn diagram of 680 mitochondrial proteins identified by mass spectrometry according to the inventory of (G).

(I) Plasma folate concentration in normal and silica-exposed mice determined by ELISA. * represents *P* < 0.05.

(J) Plasma folate concentration in healthy people and silicosis patients determined by ELISA. * represents *P* < 0.05.

(K) Demographics and plasma folate concentration of healthy people and silicosis patients.
