# Supplementary material for: Mitochondrial folate pathway regulates myofibroblast differentiation and silica-induced pulmonary fibrosis
Source: J Transl Med. 2023 Jun 6;21:365. doi: 10.1186/s12967-023-04241-0 (PMC10245413; doi:10.1186/s12967-023-04241-0)
Supplement: Supplementary file 2 — Additional file 2: Figure S2. SLC25A32 negatively regulates TGF-β induced myofibroblast differentiation, related to Fig. 2. [file 12967_2023_4241_MOESM2_ESM.docx]

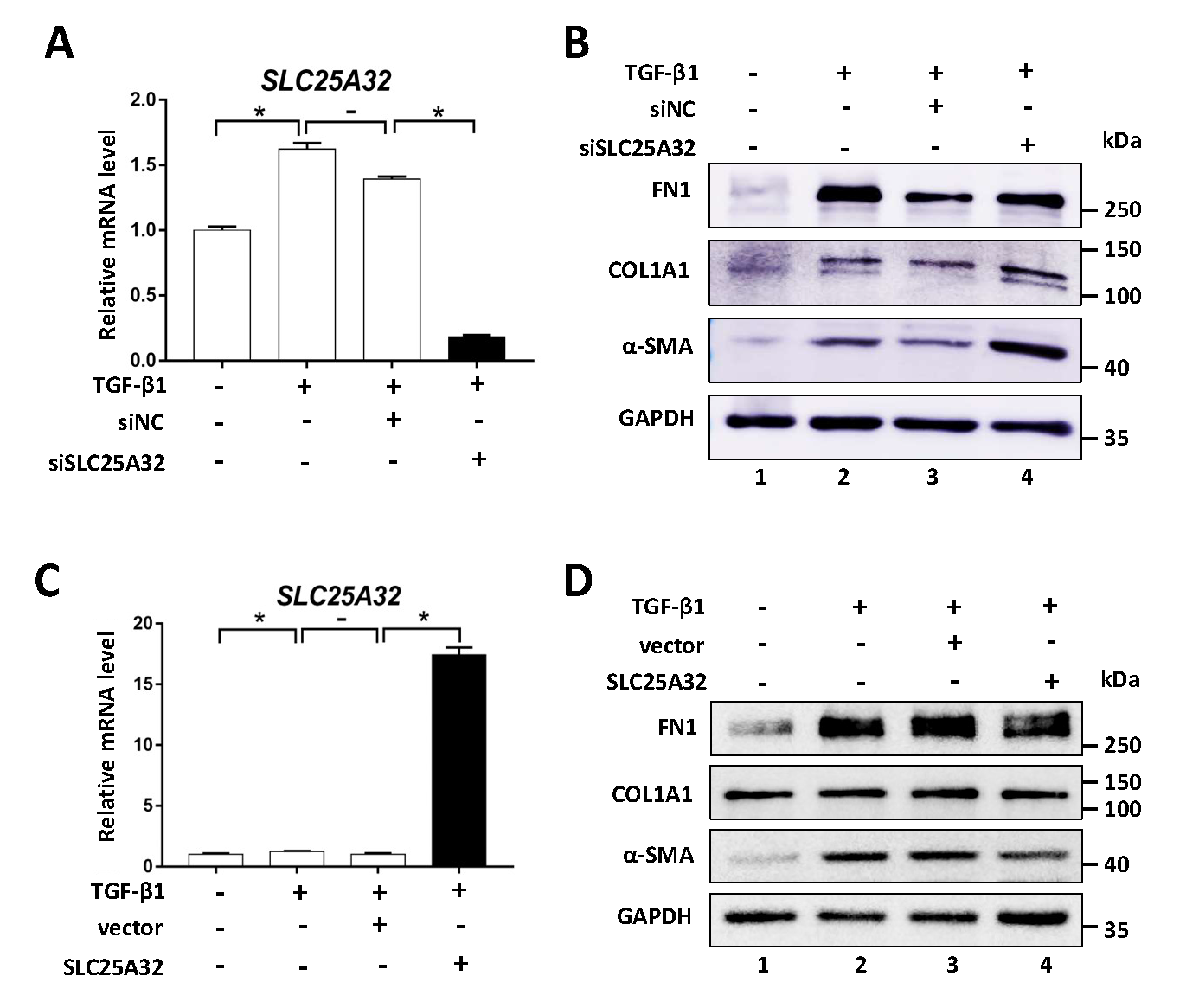


**Figure S2 SLC25A32 negatively regulates TGF-β induced myofibroblast differentiation, related to Figure 2.**

(A) The mRNA level of *SLC25A32* in cells treated with siRNA and TGF-β1 as indicated for 48 h was determined by RT-qPCR. Results are expressed as mean ± SD, n=3, * represents *P* < 0.05.

(B) Lysates from cells treated with siRNA and TGF-β1 as indicated for 48 h were analyzed by western blotting with indicated antibodies.

(C) The mRNA level of *SLC25A32* in cells transfected with indicated plasmids was determined by RT-qPCR following TGF-β1 treatment for 48 h. Results are expressed as mean ± SD, n=3, * represents *P* < 0.05.

(D) Lysates from cells transfected with indicated plasmids were analyzed by western blotting with various antibodies following TGF-β1 treatment for 48 h.
