# Supplementary material for: Mitochondrial folate pathway regulates myofibroblast differentiation and silica-induced pulmonary fibrosis
Source: J Transl Med. 2023 Jun 6;21:365. doi: 10.1186/s12967-023-04241-0 (PMC10245413; doi:10.1186/s12967-023-04241-0)
Supplement: Supplementary file 3 — Additional file 3: Figure S3. Suppressing SLC25A32 promotes TGF-β1 induced oxidative stress during myofibroblast differentiation, related to Fig. 3. [file 12967_2023_4241_MOESM3_ESM.docx]

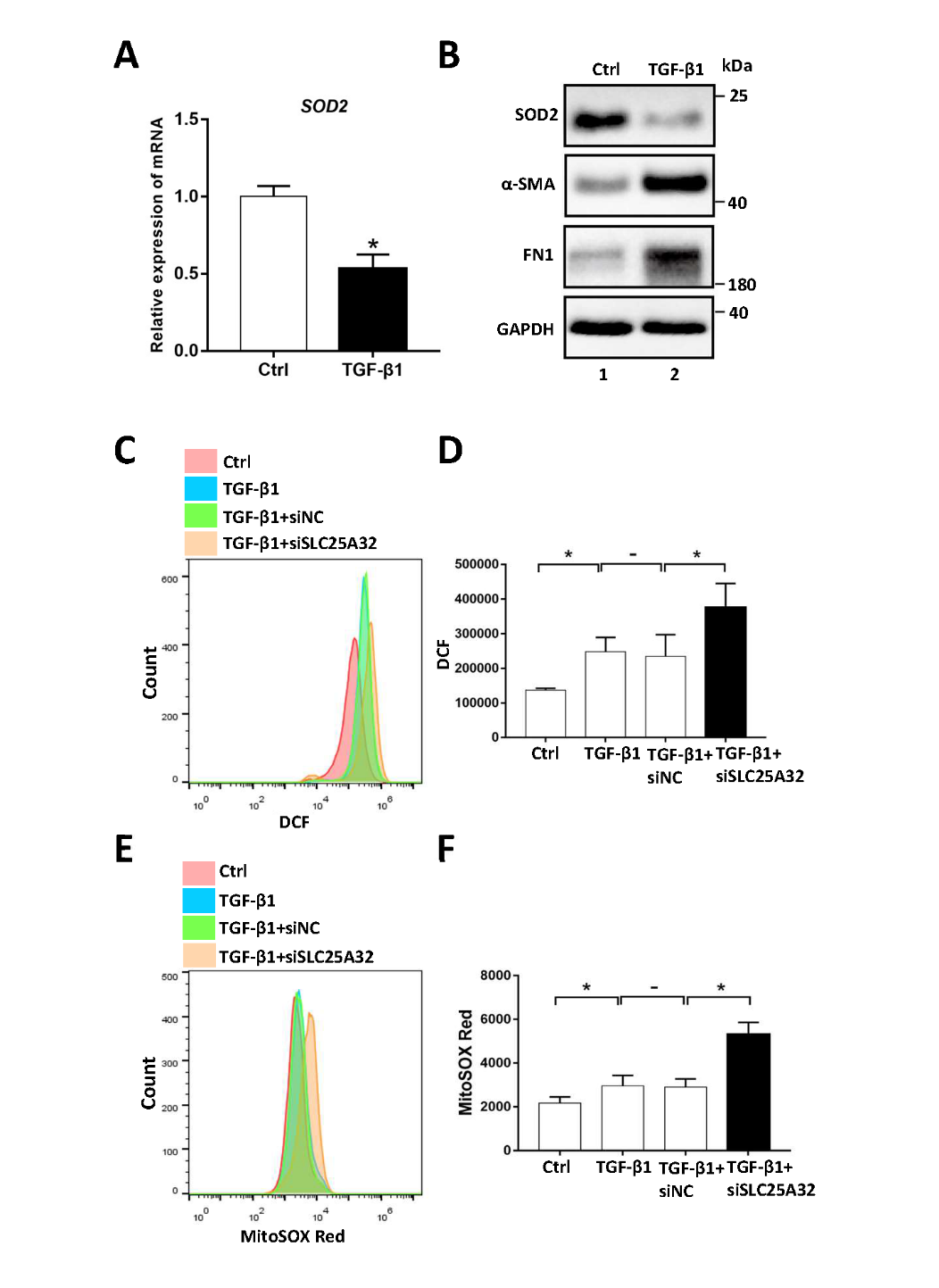


**Figure S3 Suppressing SLC25A32 promotes TGF-β1 induced oxidative stress during myofibroblast differentiation, related to Figure 3.**

(A) *SOD2* expression level in cells with or without TGF-β1 treatment for 48 h was determined by RT-qPCR. Results are expressed as mean ± SD, n=3, * represents *P* < 0.05.

(B) Western blotting of cell lysates with indicated antibodies following TGF-β treatment for 48 h.

(C)-(F) Flow cytometry analysis of total intracellular ROS (DCF) and mitochondrial ROS (MitoSOX Red) in cells treated with siRNA and TGF-β1 as indicated. Results are expressed as mean ± SD, n=3, * represents *P* < 0.05.
