# Supplementary material for: Mitochondrial folate pathway regulates myofibroblast differentiation and silica-induced pulmonary fibrosis
Source: J Transl Med. 2023 Jun 6;21:365. doi: 10.1186/s12967-023-04241-0 (PMC10245413; doi:10.1186/s12967-023-04241-0)
Supplement: Supplementary file 4 — Additional file 4: Figure S4. The effect of folate supplementation on weight and nodules in silica-treated mice, related to Fig. 4. [file 12967_2023_4241_MOESM4_ESM.docx]

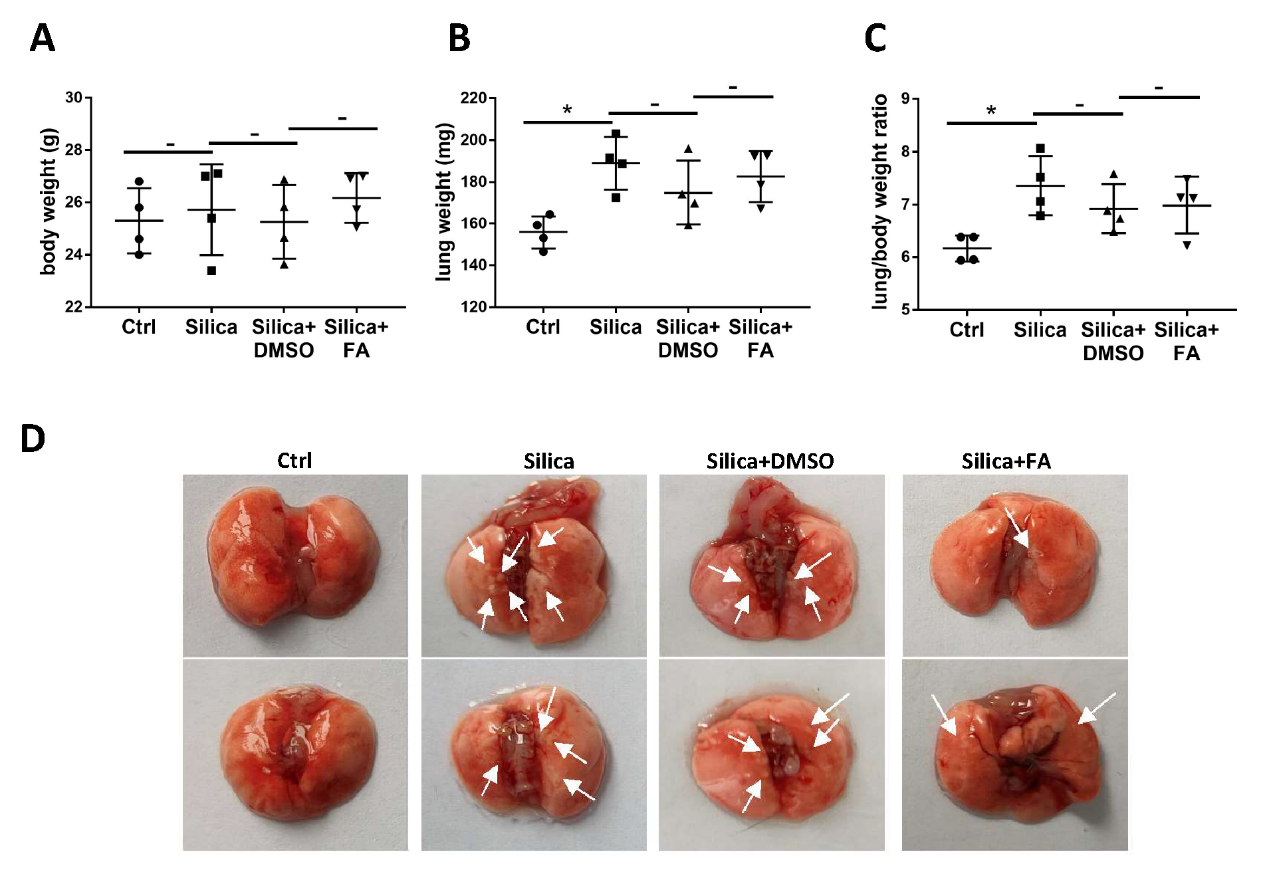


**Figure S4 The effect of folate supplementation on** **weight and nodules in silica-treated mice, related to Figure 4.**

(A)-(C) Analysis of mice body weight, lung weight and lung/body weight ratio (n=4 mice/group). * represents *P* < 0.05.

(D) The morphological observation of lungs from mice with indicated treatment. White arrows point to the megascopic nodules.
