# Supplementary material for: Mitochondrial folate pathway regulates myofibroblast differentiation and silica-induced pulmonary fibrosis
Source: J Transl Med. 2023 Jun 6;21:365. doi: 10.1186/s12967-023-04241-0 (PMC10245413; doi:10.1186/s12967-023-04241-0)
Supplement: Supplementary file 5 — Additional file 5: Figure S5. The regulation of cytosolic NADPH production pathway and mtDNA level during TGF-β induced myofibroblast differentiation. [file 12967_2023_4241_MOESM5_ESM.docx]

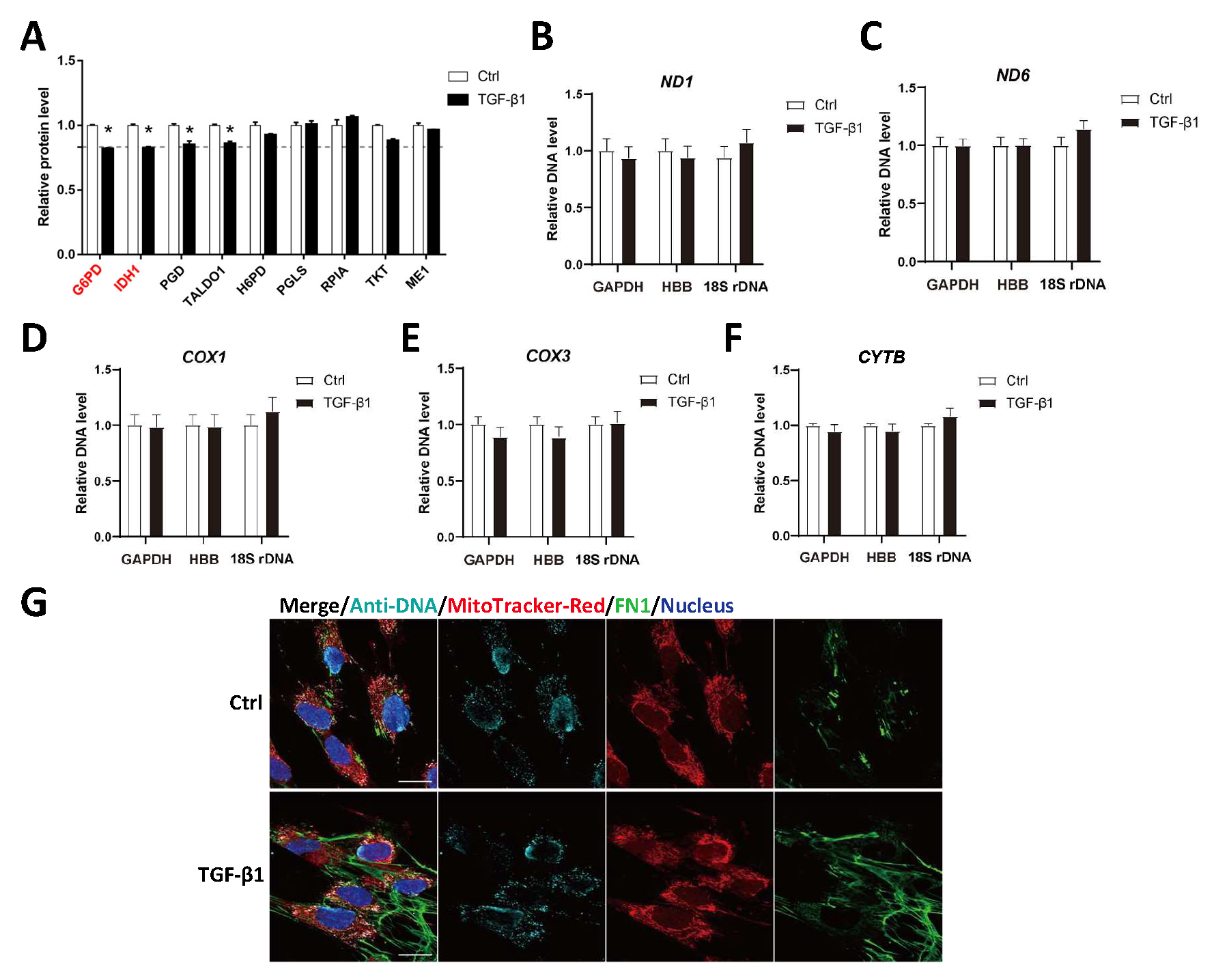


**Figure S5 The regulation of cytosolic NADPH production pathway and mtDNA level during TGF-β induced myofibroblast differentiation.**

(A) Relative abundance of proteins involved in cytosolic NADPH production pathway detected by mass spectrometry. Proteins highlighted in red were below the downregulation threshold dash line. * represents *P* < 0.05.

(B)-(F) Mitochondrial DNA levels were determined by qPCR following TGF-β treatment (normalized to GAPDH, HBB or 18S rDNA). Results are expressed as mean ± SD, n=3.

(G) Immunofluorescence of cells with indicated antibodies following TGF-β treatment and MitoTracker Red staining. Scale bar = 20 μm.
