# Supplementary material for: Mitochondrial folate pathway regulates myofibroblast differentiation and silica-induced pulmonary fibrosis
Source: J Transl Med. 2023 Jun 6;21:365. doi: 10.1186/s12967-023-04241-0 (PMC10245413; doi:10.1186/s12967-023-04241-0)
Supplement: Supplementary file 6 — Additional file 6: Figure S6. The effects of folate supplementation during myofibroblast differentiation. [file 12967_2023_4241_MOESM6_ESM.docx]

**
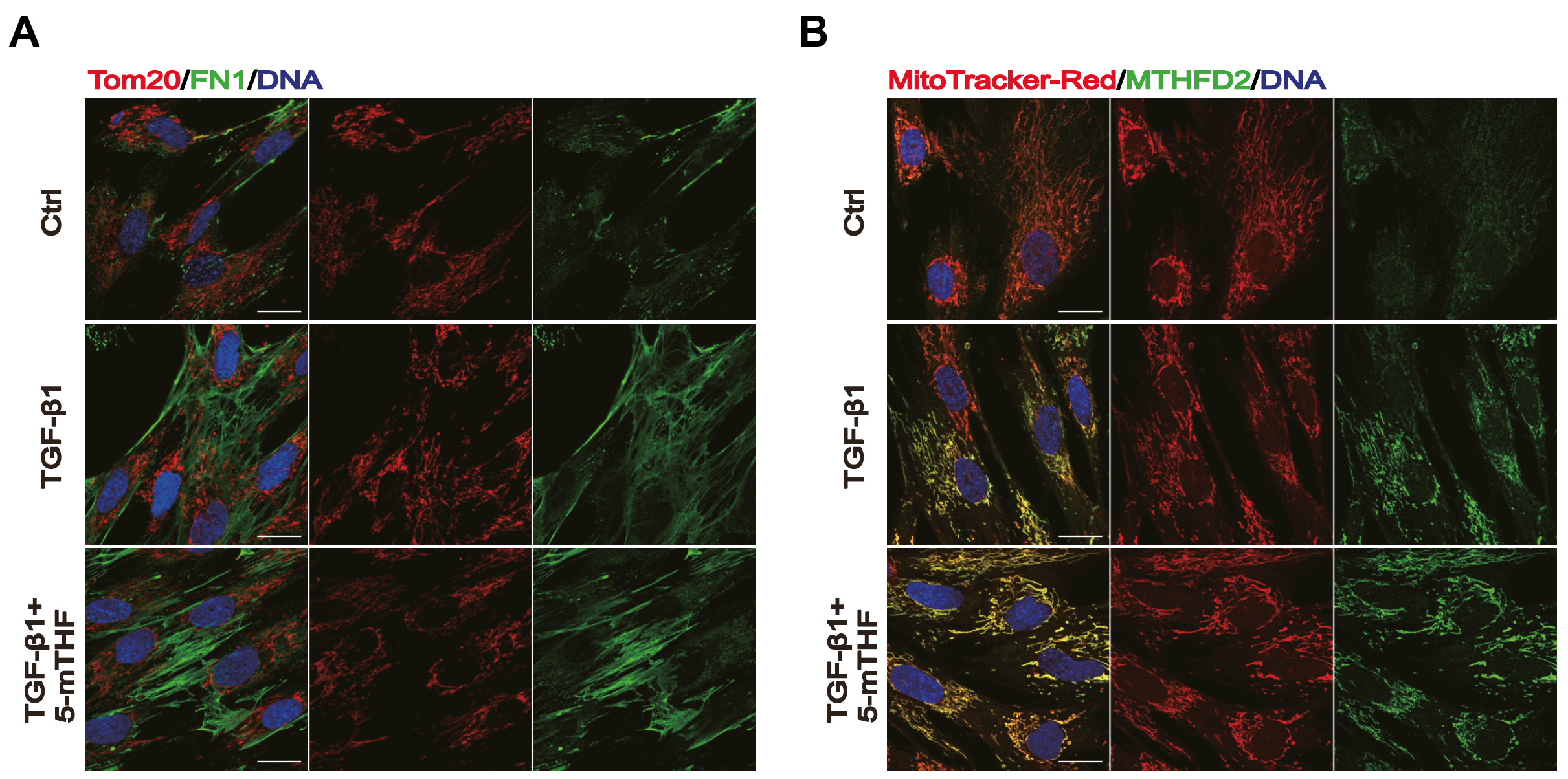
**

**Figure S6 The effects of folate supplementation during myofibroblast differentiation.**

(A)-(B) Immunofluorescence of cells with indicated antibodies and MitoTracker Red following TGF-β treatment supplemented with 5-mTHF. Scale bar = 20 μm.
