# Supplementary material for: Mitochondrial folate pathway regulates myofibroblast differentiation and silica-induced pulmonary fibrosis
Source: J Transl Med. 2023 Jun 6;21:365. doi: 10.1186/s12967-023-04241-0 (PMC10245413; doi:10.1186/s12967-023-04241-0)
Supplement: Supplementary file 7 — Additional file 7: Table S1. siRNAs and qPCR primers used in this study. [file 12967_2023_4241_MOESM7_ESM.docx]

**Table S1 siRNAs and qPCR primers used in this study.**

| **siRNA** | **Forward** | **Reverse** |
| --- | --- | --- |
| siMTHFD2 (human) | 5’-GCCUCUUCCAGAGCAUAUUTT-3’ | 5’-AAUAUGCUCUGGAAGAGGCTT-3’ |
| siSLC25A32 (human) | 5’-GAGGACAAUUCCAGUAUAUTT-3’ | 5’-AUAUACUGGAAUUGUCCUCTT-3’ |
| Negative Control (NC) | 5’-UUCUCCGAACGUGUCACGUTT-3’ | 5’-ACGUGACACGUUCGGAGAATT-3’ |
| **Gene** | **Forward** | **Reverse** |
| *GAPDH* (human) | 5′-GCACCGTCAAGGCTGAGAAC-3′ | 5′-TGGTGAAGACGCCAGTGGA-3′ |
| *FN1*(human) | 5′-ACAGAACTATGATGCCGACCAGAAG-3′ | 5′-CTGATCTCCAATGCGGTACATGA-3′ |
| *COL1A1* (human) | 5′- TAGGGTCTAGACATGTTCAGCTTTG-3′ | 5′- CGTTCTGTACGCAGGTGATTG-3′ |
| *α-SMA* (human) | 5′-ATTGCCGACCGAATGCAGA-3′ | 5′-ATGGAGCCACCGATCCAGAC-3′ |
| *SLC25A32* (human) | 5′-AGGGATTTGTTCCTGGGCTGTTTG-3′ | 5′-CTACTGTGCTCAACTGGGCTTCTG-3′ |
| *MTHFD2* (human) | 5′-CTTCGCCTTCGCCCTTTCCAC-3′ | 5′-TACCTCCTGCCGCACTTCCTG-3′ |
| *HBB* (human) | 5′-GTGCACCTGACTCCTGAGGAGA-3′ | 5′-CCTTGATACCAACCTGCCCAG-3′ |
| *18S rDNA* (human) | 5′-CGGCTACCACATCCAAGGAAG-3′ | 5′-GCTGGAATTACCGCGGCT-3′ |
| *ND1*(human) | 5′-ACGCCATAAAACTCTTCACCAAAG-3′ | 5′-TAGTAGAAGAGCGATGGTGAGAGCTA-3′ |
| *ND6* (human) | 5′-ACGCCCATAATCATACAAAGCCC-3′ | 5′-GGATTGGTGCTGTGGGTGAAA-3′ |
| *COX1* (human) | 5′-TGCCATAACCCAATACCAAACGC-3′ | 5′-CTGTTAGTAGTATAGTGATGCCAGCAGCTAGG-3′ |
| *COX3* (human) | 5′-CGATACGGGATAATCCTATTTATTACCTCAG-3′ | 5′-CAGGTGATTGATACTCCTGATGCGA-3′ |
| *CYTB* (human) | 5′-CGCCTGCCTGATCCTCCAA-3′ | 5′-AGGCCTCGCCCGATGTGTAG-3′ |
| *Gapdh* (mouse) | 5′-GGTTGTCTCCTGCGACTTCA-3′ | 5′-TGGTCCAGGGTTTCTTACTCC-3′ |
| *Fn1*(mouse) | 5′-CTATAGGATTGGAGACACGTGG-3′ | 5′-CTGAAGCACTTTGTAGAGCATG-3′ |
| *Col1a1* (mouse) | 5′-TGAACGTGGTGTACAAGGTC-3′ | 5′-CCATCTTTACCAGGAGAACCAT-3′ |
| *α-Sma* (mouse) | 5′-GCGTGGCTATTCCTTCGTGACTAC-3′ | 5′-CGTCAGGCAGTTCGTAGCTCTTC-3′ |
| *Slc25a32* (mouse) | 5′-CCGTAGCAGCAACATACCCGTATC-3′ | 5′-CACCGATGCCTTCTTTCCTCCAC-3′ |
| *Mthfd2* (mouse) | 5′-CAAGGAAGGAGCAGCGGTCATC-3′ | 5′-ACCAGGGACGGGAGTGATATAACC-3′ |
| *Tgfb1* (mouse) | 5′-CCAGATCCTGTCCAAACTAAGG-3′ | 5′-CTCTTTAGCATAGTAGTCCGCT-3′ |
